# Supplementary material for: 3D cell culture stimulates the secretion of in vivo like extracellular vesicles
Source: Sci Rep. 2019 Sep 10;9:13012. doi: 10.1038/s41598-019-49671-3 (PMC6736862; doi:10.1038/s41598-019-49671-3)
Supplement: Supplementary file 1 — SUPPLEMENTARY INFO [file 41598_2019_49671_MOESM1_ESM.docx]

**3D cell culture stimulates the secretion of in vivo like extracellular vesicles**

**Sirisha Thippabhotla^1^, Cuncong Zhong^1,2^, Mei He^2,3,4^***

1. Department of Electrical Engineering and Computer Science, University of Kansas, Lawrence, Kansas 66045, USA
2. Bioengineering Research Center, University of Kansas, Lawrence, Kansas 66045, USA
3. Department of Chemical and Petroleum Engineering, University of Kansas, Lawrence, Kansas 66045, USA
4. Department of Chemistry, University of Kansas, Lawrence, Kansas 66045, USA

* meih@ku.edu

# Methods

# EV Isolation and RNA/DNA Extraction:

# EV isolation steps are illustrated in SI Figure s1. For collecting 2D cultured medium used to characterize EV secretion dynamics, several time intervals were performed according to the confluence behavior (90% confluence in 48 hrs): 6, 12, 24, 36, 48, and 60 hours post subculture. To remove the cell debris, the medium was centrifuged at 3,000g for 15 mins within 4 °C. To collect the supernatant carefully ready for EV isolation, the supernatant was spined at 10,000g for 20 mins, and the debris at the bottom was discarded. For collecting 3D cultured medium used to characterize EV secretion dynamics, below time intervals were performed according to the 3D cell confluence behavior (90% confluency in 11 days): 5, 7, 9, 11, 13 days post subculture. For recovering 3D spheroids, the scaffolding hydrogels were broken mechanically by pipetting up and down, and then transferred into a 15 mL tube for centrifuging at 600 g for 10 min at room temperature for collecting cell pellet. ~0.5 mL 0.25% trypsin (Gibco, 25200-056) was added into the pellet, resuspended gently, and then incubated at 37 °C for 5 min. The trypsin digestion was stopped by adding 0.5 mL MEM complete medium, and the cell pellet was ready for use. For collecting 3D culture derived medium, after breaking scaffolding hydrogels by pipetting up and down, the entire solution was vortexed for 2 minutes and transferred into a 15 mL tube for centrifuging at 600 g for 10 min at room temperature. The supernatant was collected carefully for EV isolation. We use centrifugation protocols developed in our lab to collect EVs-containing supernatant detailed in SI Figure s1. The collected supernatants were subject to a filtration process developed in our lab. The 0.22 µm filter (Millipore Express PLUS (PES) membrane) was conditioned with 10% (v/v) exosome-depleted fetal bovine serum for 2 minutes, then introduce prepared supernatants for filtration. The 10% exo-depleted FBS conditioning could reduce the trap of small EVs when filtration. We did compare the EV particle numbers before and after filtration using NTA analysis and did not see much difference, which is also consistent with the reported filtration method for ensuring the purity of smaller EVs[1]. Afterward, we combine Qiagen ExoRNeasy kit which contains exoEasy spin filtration column. The exoEasy spin column was used for spinning at 500 g for 1min. The flow-through medium was discarded and spin again for 1 min at 3,200 g. ~10 mL XWP buffer was added to the column and spin for 10 min at 3,200 g to wash the trapped exosomes in the membrane of the spin column for following downstream RNA or DNA extraction.

#
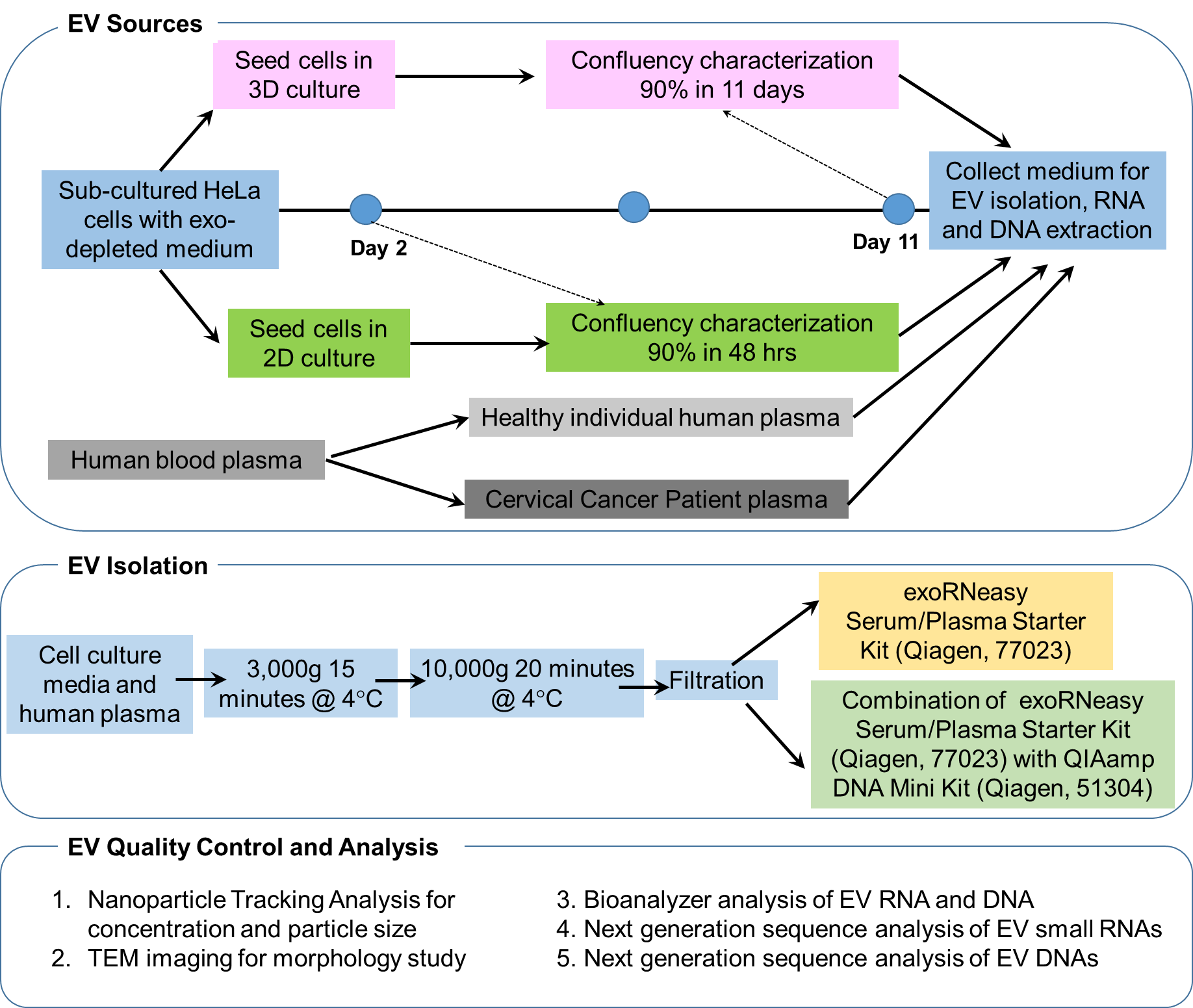


**Figure s1**. Scheme of the experimental flow for EV production, isolation, characterization, and data analysis. Quality control techniques (NTA, TEM, and Bioanalyzer) were performed on the isolated EVs prior to next-generation sequencing of small RNAs and DNAs.

# Characterization of 3D HeLa Cell Confluency Behavior:

For characterizing 3D cell confluency behavior, we count the 3D cultured live cells at certain duration (6 hours after seeding, 2 days, 3 days, 5 days, 6 days and 8 days). The hydrogel can be broken down by pipetting up and down of culture for several times. Then the spheroids were suspended and transferred to 15 mL tube for rinsing with 2 mL of media twice. Mix 8 mL more media into the tube. Centrifuge at 250 g for 5 minutes. Use micro-pipette at 50 μL to remove all media from cell pellet but not to disturb the cell pellet. Resuspend the cell pellet in 50 μL Trypsin and mix well for 5 minutes, and then add 50 uL of Trypsin inhibitor in the mixture. Re-measure the total volume if possible (usually the volume will be 110 uL from residual media and cells). Add an equal amount of Trypan blue and use 10 uL to count cells (Cell counting chamber, Fisher).

**
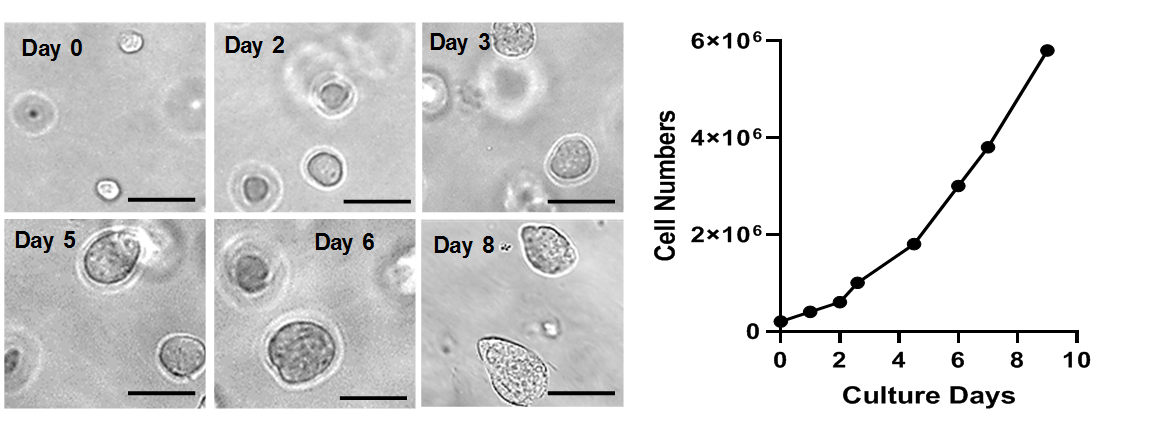
**

**Figure s2.** Time-dependent HeLa cell 3D culture. Cell culture conditions according to the method were described in the experimental section. Bright-field microscopic images (left) represent the 3D cell morphology cultured from 0 day (6 hours after seeding), 2 days, 3 days, 5 days, 6 days, to 8 days, respectively. The initial seeding density is ~8×10^4^ cells/mL. The scale bar is 80 µm. 3D cultured live HeLa cells are counted using the method described above along different culture durations for calibrating and estimating total cell numbers and growth rate (calibration curve in right hand).

**Quantitative Real-time PCR for Validating NGS Data**

TaqMan Advanced miRNA assays are delivered in a single tube containing the specific pre-formulated TaqMan Assay (TaqMan MGB probe, and forward and reverse primers). miR-Amp reagents are included in the TaqMan Advanced miRNA cDNA Synthesis Kit, and universal primers and master mix was used to uniformly increase the amount of cDNA for each target. We used six assay kits for selected miRNAs, including Hsa-miR-125b-1-3p, Hsa-miR-208a-5p, Hsa-miR-450b-3p, Hsa-miR-1229-3p, Hsa-miR-1284, Hsa-miR-1909-3p. We set up and run the real-time PCR instrument with the appropriate PCR thermal cycling conditions and selected the fast cycling mode for all instruments with 40 cycles.

**
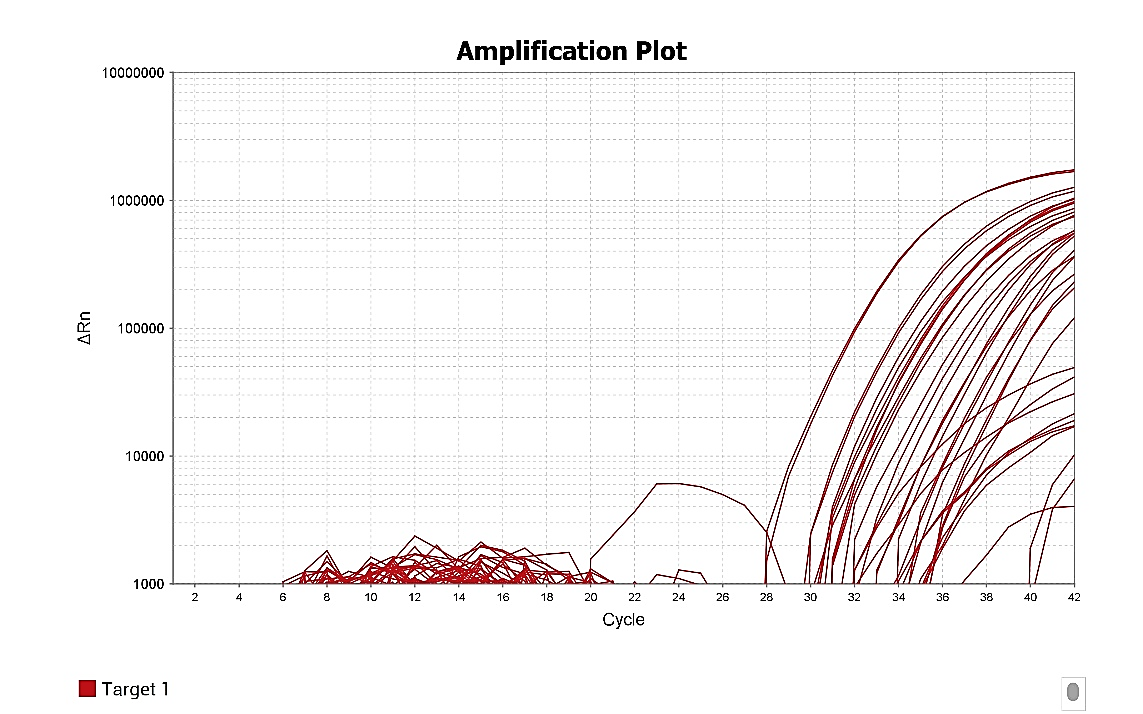
**

**Figure s3.** The amplification plots depict the qPCR cycles of six selected miRNAs with repeats.

**Table s1. Top pathways with 10-fold change for miRNAs significantly enriched in EVs from their parent cells (2D system)**

| ***p*-value** | **Pathways** | **External IDs** | **Gene IDs** |
| --- | --- | --- | --- |
| 4.05583E-16 | ABC transporters - Homo sapiens (human) | path:hsa02010 | 19; 22; 5825; 9429; 10349; 10350; 64241; 9619 |
| 7.94085E-13 | ABC-family proteins mediated transport | R-HSA-382556 | 5825; 23; 10350; 9619; 10349; 64241; 22 |
| 2.46769E-11 | ABC transporters in lipid homeostasis | R-HSA-1369062 | 10350; 5825; 10349; 9619; 64241 |
| 7.83492E-08 | Transport of small molecules | R-HSA-382551 | 10350; 64241; 19; 5825; 23; 22; 10349; 9619; 9429 |
| 1.46137E-05 | Nuclear Receptors in Lipid Metabolism and Toxicity | WP299 | 9619; 5825; 19 |
| 0.000543634 | Alanine Aspartate Asparagine metabolism | None | 16; 18 |
| 0.000838456 | Statin Pathway | WP430 | 64241; 19 |
| 0.001466377 | Fat digestion and absorption - Homo sapiens (human) | path:hsa04975 | 19; 64241 |
| 0.00217437 | Cholesterol metabolism - Homo sapiens (human) | path:hsa04979 | 19; 64241 |
| 0.002816538 | C21-steroid hormone biosynthesis and metabolism | C21-steroid hormone biosynthesis and metabolism | 9619; 64241 |
| 0.004099225 | Plasma lipoprotein assembly, remodeling, and clearance | R-HSA-174824 | 9619; 19 |
| 0.004334931 | Bile secretion - Homo sapiens (human) | path:hsa04976 | 9429; 64241 |

**Table s2. Top pathways with 10-fold change for miRNAs significantly enriched in EVs from their parent cells (3D system)**

| ***p*-value** | **Pathways** | **External IDs** | **Gene IDs** |
| --- | --- | --- | --- |
| 1.34641E-11 | ABC transporters - Homo sapiens (human) | path:hsa02010 | 64137; 4363; 19; 9429; 10350; 9619 |
| 9.24674E-09 | ABC-family proteins mediated transport | R-HSA-382556 | 23; 4363; 10350; 9619; 64137 |
| 1.5424E-06 | ABC transporters in lipid homeostasis | R-HSA-1369062 | 10350; 9619; 64137 |
| 1.16293E-05 | Transport of small molecules | R-HSA-382551 | 10350; 64137; 19; 23; 4363; 9619; 9429 |
| 3.43433E-05 | Methotrexate Pathway (Brain Cell), Pharmacokinetics | PA165816270 | 4363; 9429 |
| 3.43433E-05 | Paclitaxel Action Pathway | SMP00434 | 9429; 4363 |
| 3.43433E-05 | Docetaxel Action Pathway | SMP00435 | 9429; 4363 |
| 0.000126967 | Irinotecan Pathway | WP229 | 4363; 9429 |
| 0.000126967 | Taxane Pathway, Pharmacokinetics | PA154426155 | 4363; 9429 |
| 0.000126967 | Methotrexate Pathway, Pharmacokinetics | PA165816349 | 4363; 9429 |
| 0.000126967 | Irinotecan Pathway, Pharmacokinetics | PA2001 | 4363; 9429 |
| 0.000220692 | Doxorubicin Metabolism Pathway | SMP00650 | 4363; 9429 |
| 0.000248086 | Doxorubicin Pathway (Cancer Cell), Pharmacodynamics | PA165292163 | 4363; 9429 |
| 0.000248086 | Lamivudine Metabolism Pathway | SMP00649 | 4363; 9429 |
| 0.000248086 | Lamivudine Pathway, Pharmacokinetics/Pharmacodynamics | PA165860384 | 9429; 4363 |
| 0.000373399 | Doxorubicin Pathway, Pharmacokinetics | PA165292177 | 4363; 9429 |
| 0.000408644 | Irinotecan Action Pathway | SMP00433 | 4363; 9429 |
| 0.000408644 | Irinotecan Metabolism Pathway | SMP00600 | 4363; 9429 |
| 0.000445447 | Pathway_PA165986194 -need delete | PA165986194 | 4363; 9429 |
| 0.000445447 | Acetaminophen Pathway, Pharmacokinetics | PA165986279 | 4363; 9429 |
| 0.0006988 | Acetaminophen Metabolism Pathway | SMP00640 | 4363; 9429 |
| 0.000952126 | Nuclear Receptors in Lipid Metabolism and Toxicity | WP299 | 9619; 19 |
| 0.003656373 | Plasma lipoprotein assembly, remodeling, and clearance | R-HSA-174824 | 9619; 19 |

**Reference:**

1. Gheinani AH, Vogeli M, Baumgartner U, Vassella E, Draeger A, Burkhard FC, Monastyrskaya K: **Improved isolation strategies to increase the yield and purity of human urinary exosomes for biomarker discovery**. *Sci Rep* 2018, **8**(1):3945.
